# A Silent Killer: Left Main Coronary Artery Disease in Gastrointestinal Bleed

**DOI:** 10.7759/cureus.15988

**Published:** 2021-06-28

**Authors:** Hesham Afify, Volodymyr Oliynyk, Floyd Burke

**Affiliations:** 1 Internal medicine, University of Central Florida/HCA Healthcare Graduate Medical Education, Orlando, USA; 2 Internal Medicine, University of Central Florida/HCA Healthcare Graduate Medical Education, Orlando, USA; 3 Cardiology, Orlando Veterans Affairs (VA) Medical Center at Lake Nona, Orlando, USA; 4 Medicine, University of Central Florida College of Medicine, Orlando, USA

**Keywords:** left main coronary artery disease (lmcad), electrocardiogram (ecg), augmented lead avr, st segment elevation, acute gastrointestinal bleed, type 2 myocardial infarction

## Abstract

Left main coronary artery disease (LMCAD) is defined as more than 50% angiographic arterial narrowing and has been demonstrated in nearly 5% of all patients undergoing coronary angiography. It carries an extremely high risk for cardiovascular morbidity and mortality as it impacts more than two-thirds of the left ventricle. Prediction of LMCAD in the right clinical setting is important for the selection of the proper treatment strategies. Typical ECG characteristics are ST elevation (STE) in lead augmented vector right (aVR-STE) of more than 0.5 mV accompanied by ST depression (STD) notably in leads I, II, and V4-6 or STE in aVR ≥ V_1_. Furthermore, the presence of aVR-STE is associated with worse outcomes and careful evaluation and close monitoring are warranted. However, not every aVR-STE is an acute occlusion of the left main coronary artery (LMCA), as acute occlusion is a catastrophic event. aVR-STE can also be associated with severe triple-vessel disease or diffuse subendocardial ischemia.

## Introduction

Left main coronary artery disease (LMCAD) is a catastrophic disease that presents with either occlusion or chronic obstruction. The ECG is of ultimate importance and has high sensitivity and specificity in diagnosing/predicting LMCAD [1]. Patients with LMCAD need close monitoring and early recognition of the ECG changes by the treating clinician. These ECG changes are associated with worse outcomes and represent diffuse myocardial ischemia [2].

## Case presentation

**Figure 1:**
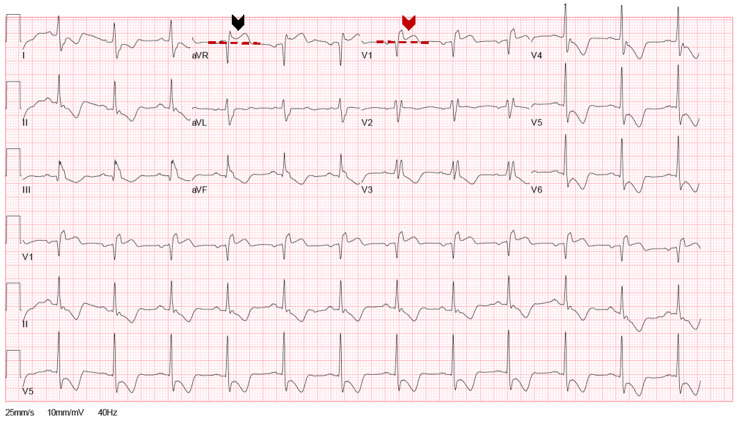
The 12-lead ECG at admission. A 12-lead ECG shows normal sinus rhythm with complete right bundle branch block (RBBB), ST-segment elevation in both augmented vector right (aVR) (black arrow) and V1 (red arrow). The degree of ST elevation in lead aVR is higher than V1. ST-segment depression was noted in leads I, II, and V4-V6 as well as a T-wave inversion in the inferolateral leads.

A 67-year-old man presented to the ED with a chief complaint of fatigue, dark stool, dyspnea, and chest pain on exertion. He was found to be afebrile on clinical examination, with a respiratory rate of 22, oxygen saturation of 100% on room air, and blood pressure of 96/44 mm Hg. Heart rate was 75 beats per minute. He had a known history of coronary artery disease (CAD), complete total occlusion (CTO) of the right coronary artery (RCA), and drug-eluting stent to the left anterior descending (LAD). Additionally, the patient had a right internal carotid artery stenting on dual antiplatelet therapy, non-insulin-dependent diabetes mellitus, chronic stage 3A kidney disease, and iron deficiency anemia. Admission laboratory studies revealed severe anemia with hemoglobin (Hgb) of 5.3 g/dl, mean corpuscular volume (MCV) of 109 fL, and troponin I (TnI) of 0.34 ng/mL. ECG on admission is shown in Figure 1.

The ECG shows normal sinus rhythm at a rate of 72 beats per minute with complete right bundle branch block (RBBB), ST-segment elevation in both aVR and V1. The degree of ST elevation in lead aVR is higher than V1. ST-segment depression in leads I, II, and V4 to V6 and a T-wave inversion in the inferolateral leads suggest LMCA or proximal LAD artery disease. In comparison with previous ECGs, the above findings were new. The patient was diagnosed with type 2 myocardial infarction (T2MI) provoked by anemia based on history, presentation, and ECG changes.

Clinical Course

The patient was admitted to the ICU for stabilization, blood transfusion, and further medical evaluation. He received three units of packed red blood cells. Gastroenterology consultation was requested for further diagnostic evaluation. Esophagogastroduodenoscopy revealed one non-bleeding angiodysplastic lesion in the stomach treated with argon plasma coagulation. The patient’s hemoglobin stabilized after blood transfusion and there was no further clinical bleeding. Given the initial presentation, a cardiac workup was performed. Echocardiography revealed mildly increased left ventricular thickness, grade II diastolic dysfunction, ejection fraction of 50%, and no left ventricular regional wall motion abnormalities. In comparison with previous ECGs, the above findings were new. As a result of the abnormal ECG and elevated TnI level, the patient was referred for coronary angiography. Severe ostial left main disease with ostial LAD disease was found along with the known CTO of the RCA. The patient was referred for surgical myocardial revascularization.

## Discussion

The left ventricle and interventricular septum are supplied mostly by the LMCA and its two main branches: the LAD and the left circumflex coronary arteries. LMCAD carries an extremely high risk for cardiovascular morbidity and mortality as it impacts more than two-thirds of the left ventricle. Significant LMCAD is defined as more than 50% angiographic arterial narrowing and has been demonstrated in nearly 5% of all patients undergoing coronary angiography [3]. The most common cause for LMCAD is atherosclerosis and it is associated with atherosclerotic burden in other coronary arteries. The incidence of LMCAD is expected to increase with heightened predominance in individuals carrying cardiovascular risk factors and in the elderly population [4]. Without revascularization, patients with significant LMCAD have an estimated three-year survival rate of 37% [5]. Prediction of LMCAD in the right clinical setting is important for the selection of the proper treatment strategies. The ECG is important and certain ECG characteristics have been identified for LMCAD. Typical ECG characteristics are aVR-STE of more than 0.5 mV accompanied with ST depression (STD) in leads I, II, and V4-6 [1]. Furthermore, STE in aVR greater than or equal to V1 has a high sensitivity (81%) and specificity (80%) for LMCAD [1]. Acute occlusion of the LMCA is a catastrophic event that most often leads to significant hemodynamic compromise or sudden cardiac death. However, total obstruction of LMCA is very rare. In a single-center retrospective study of patients with isolated aVR-STE with multi-lead STD presenting to the ED, only 10% of the patients had an acutely occluded vessel but none had an acutely occluded left main or LAD [6]. Clinicians should be aware that aVR-STE can also be associated with severe triple-vessel disease [7] and diffuse subendocardial ischemia [2]. The presence of aVR-STE during exercise testing provides evidence of the association between aVR-STE and subendocardial ischemia [8]. Furthermore, aVR-STE carries a prognostic value in certain clinical situations; aVR-STE > 1 mm is associated with a higher 30-day mortality rate in ST-elevation myocardial infarction (STEMI) regardless of the infarct location, as was demonstrated by the HERO-2 study [9]. Additionally, the resolution of these ECG changes was associated with improved patient mortality [9]. aVR-STE is estimated to be present in half of the patients with inferior wall myocardial infarction. Such incidence is associated with a 27% increase in mortality [10]. In non-ST segment elevation myocardial infarction (NSTEMI), the presence of aVR-STE is associated with higher in-hospital death, congestive heart failure, and recurrent ischemia [11]. Thus, the presence of aVR-STE requires careful evaluation and close monitoring. The mechanism of aVR-STE is unclear, as aVR is the augmented unipolar right arm lead and is considered the window into the heart cavity from the right shoulder. Thus, transmural ischemia in the basal portion of the interventricular septum or global subendomyocardial ischemia could lead to aVR-STE. Moreover, aVR is electrically opposite to lateral limb and pericardial leads (I, II, aVL, and V4-6), and aVR-STE can represent the reciprocal changes to ST-segment depression in these leads [12]. Our patient was considered to have T2MI provoked by anemia based on the patient's history and presentation. T2MI is a clinical diagnosis defined by myocardial injury secondary to supply-demand mismatch without plaque disruption [13]. The prevalence of concomitant CAD in patients with T2MI is estimated to be between 36% and 78% [14]. The intensity of supply-demand discrepancy necessary to induce ischemia is determined by the severity of concomitant CAD. Management of T2MI is mainly conservative and focuses on the precipitating factor. The presence of typical ECG changes for LMCAD in our patient led to the pursuit of coronary angiography for further delineation of the coronary anatomy. Subendocardial ischemia secondary to severe LMCAD is the most likely explanation for the ECG changes provoked by GI bleed and severe anemia that led to a clinically significant supply-demand mismatch. This is evident by the widespread STD in leads I, II, and V4 to V6.

## Conclusions

aVR-STE exists in multiple clinical scenarios. It can be due to either significant occlusion of the left main or proximal LAD, severe triple-vessel disease, or diffuse subendocardial ischemia. The left ventricle and interventricular septum are supplied mainly by the LMCA. Prediction of LMCAD occlusion or chronic obstruction is important, as it carries an extremely high risk for cardiovascular morbidity and mortality. Typical ECG characteristics for LMCAD are aVR-STE of more than 1 mm accompanied with STD notably in leads I, II, and V4-6. Moreover, STE in aVR ≥ V1 has high sensitivity and specificity for LMCAD. Augmented lead VR carries important diagnostic and prognostic value for the clinician. Thus, understanding the pathophysiology and recognizing aVR-STE in the right scenario is helpful in making a prompt diagnosis of potential left main or multivessel CAD.
